# Antipruritic Effects of Botulinum Neurotoxins

**DOI:** 10.3390/toxins10040143

**Published:** 2018-03-29

**Authors:** Parisa Gazerani

**Affiliations:** Department of Health Science and Technology, Aalborg University, Frederik Bajers Vej 7A2, A2-208, 9220 Aalborg East, Denmark; gazerani@hst.aau.dk; Tel.: +45-9940-2412

**Keywords:** botulinum neurotoxins, itch, pruritus, antipruritic, clinical, experimental

## Abstract

This review explores current evidence to demonstrate that botulinum neurotoxins (BoNTs) exert antipruritic effects. Both experimental and clinical conditions in which botulinum neurotoxins have been applied for pruritus relief will be presented and significant findings will be highlighted. Potential mechanisms underlying antipruritic effects will also be discussed and ongoing challenges and unmet needs will be addressed.

## 1. Introduction

Botulinum neurotoxins (BoNTs) are protein neurotoxins that are produced by anaerobic, spore-forming bacteria of the *Clostridium* genus, including *Clostridium botulinum*, *Clostridium butyrricum*, *Clostridium barati,* and *Clostridium argentinensis* [1]. An updated review of biology, pharmacology, and toxicology of BoNTs can be found in M. Pirazzini et al.’s excellent recent review [2]. Since its first therapeutic use in humans in the 1980s [3], clinical use of BoNTs has significantly increased [4]. BoNTs have been used for therapeutic purposes in a diverse range of medical conditions, such as ophthalmology, neurology, gastroenterology, urology, and psychiatry [2,4,5,6]. Advanced understanding of the mechanism of action of BoNTs has led to increasing use of these molecules with novel, unique, and desirable pharmacological properties [2]. BoNTs have also been tested in many dermatological conditions, several of which with off-label uses [7,8]. This review focuses on the antipruritic effects of BoNTs as presented in clinical and experimental conditions which specifically addressed potential itch-relieving mechanisms. The aim is to highlight the value of BoNTs in an expanded evaluation as potential antipruritic agents in future practice. It is worth mentioning that one case [9] in the literature presented that an application of OnabotulinumtoxinA (100 U) provoked itchiness in a patient when it was used for neuromuscular pain. In that instance, pruritus was treated with oral hydroxyzine and camphor-menthol topical lotion [9]. 

## 2. Pruritus

Itchiness (pruritus) is a common unpleasant sensation that elicits a desire to scratch [10]. Acute itch [11] serves as a warning and self-protective mechanism to prevent potentially harmful irritations. However, chronic itch is a challenging and significant clinical problem [12] which is often associated with skin diseases, systemic diseases, metabolic disorders, and psychiatric disorders [13]. Although scratching temporarily relieves acute itch, persistent itch-scratch cycles often exacerbate skin problems, disrupt sleep, and reduce the quality of life in chronic itch patients [14]. Recent studies have documented a high prevalence of chronic pruritus (13–17%) with a lifetime prevalence of 22–26% [15]. On the basis of international consensus, a threshold of six weeks has been set for the definition of chronic pruritus. Chronic pruritus is a major challenge to overcome [16] and requires interdisciplinary cooperation despite many interfering factors. Successful treatment usually involves dermatologists, internists, general practitioners, neurologists, gynecologists, and psychiatrists [16]. An understanding of the molecular mechanisms underlying itch has advanced the identification of itch-specific pathways and transmitters for selective targeting [17].

Itch can broadly be categorized as either histaminergic and non-histaminergic [18]. Histamine is released by mast and epithelial cells and binds to H1–H4 receptors, leading to the activation of downstream target molecules within sensory neurons [17,19,20]. Histamine has been identified as the main mediator of itch in several pruritic conditions, such as urticaria and allergic diseases. Antipruritic treatment strategies [12] have been successfully used to target histaminergic pathways [21]. 

Non-histaminergic itch has attracted more attention in recent years because many chronic itch conditions are resistant to antihistamines, necessitating the need to consider alternative treatments. Cowhage (*Mucuna pruriens*) is a tropical legume known to cause itch, pricking, stinging, and burning sensations that otherwise do not respond to antihistamines. This characteristic has made cowhage a useful tool for studying non-histaminergic mechanisms underlying itch. When cowhage spicules are inserted, the cysteine protease mucunain is released. When the mucanin reaches the nerve endings of primary sensory neurons in the epidermis, it activates protease-activated receptors (PAR) 2 and 4, which are members of the G protein-coupled receptor family [22]. Interestingly, PAR2 and tryptase (an endogenous PAR2 agonist, the most abundant secretory granule-derived serine proteinase released by mast cells causing itch), have been found highly elevated in patients with atopic dermatitis (AD) [23]. Mas-related G protein-coupled receptors (Mrgprs) are involved in the response to non-histaminergic pruritogens [24]. MrgprA3 is expressed in a sub-population of sensory neurons known as peptidergic C-fibers and encodes pruritic effects of chloroquine, which is an antimalarial drug [25,26]. These receptors [27,28] are also responsive to histamine, bovine adrenal medulla 8–22 (BAM8–22), cowhage spicules, and capsaicin. Interestingly, mice lacking MrgprA3 neurons are resistant to pruritogens such as histamine, BAM8–22, SLIGRL, α-methyl-5HT, ET-1, and chloroquine [26]. However, MrgprA3-ablated mice scratch in response to β-alanine [29]. Therefore, MrgrpA3 positive sensory neurons are different from those neurons responding to β-alanine for itch. MrgprD is the receptor activated by β-alanine [30], and mice lacking MrgprD do not scratch following an intradermal injection of β-alanine [29]. Transient receptor potential (TRP) channels are also involved in itch. Histaminergic itch transmission through TRPV1 has been reported [31]. TRPA1 is a downstream target of MrgprA3 and MrgprC11. Ablation of TRPA1 blocks itch in a dry skin mouse model of chronic itch. Mice lacking TRPA1 exhibit no scratch following subcutaneous injections of chloroquine and BAM8–22, but do scratch in response to α-methyl-5HT [17,32]. Voltage-gated sodium channel (NaV) 1.7 has also been found to mediate itch. A monoclonal antibody targeting NaV 1.7 could abolish both acute and chronic itch in mice [33]. 

Immune cells of skin interact with nerve endings and play an important role in pathological itch. Cytokines released from T helper 2 cells are found elevated in several pruritus conditions [34]. Interleukin-31 (IL-31) is known to play a role in AD [35]. Intradermal injection of IL-31 in mice provokes scratching [36] that is also correlated with elevated expression of IL-31RA in the DRG (dorsal root ganglion) [37].

Taken together, novel findings on peripheral receptors and mediators [38] present that itch is mediated by several different subpopulations of primary sensory neurons. Some itch-provoking substances activate overlapping populations of neurons, while others only activate distinct populations (e.g., chloroquine vs. β-alanine; MrgprA3 vs. MrgprD). Depending on pruritogen, method of delivery, and species, itch-related responses are variable [17]. One must also consider that a cross-talk exists between the neurons and immune cells in the skin [38].

In addition to advancements in peripheral mechanisms of itch, several hypotheses have been proposed for central mechanisms of itch [17,38]. Gastrin-releasing peptide (GRP) and GRP receptors were the first central components of itch identified in the spinal cord [39]. Ablation of inhibitory interneurons (B5-I) in mice resulted in the development of skin lesions and scratching in mice [40]. B5-I interneurons are activated following certain transient receptor potential (TRP) channels signaling and release dynorphin that can block itch signaling [41]. Spinal interneurons that express neuropeptide Y also exist which mediate mechanical itch [42]. Cross-talk between neurons and central glia has also been suggested in modulating itch [43]. For example, in a mouse model of contact dermatitis and AD, spinal reactive astrogliosis has been reported [44]. Toll-like receptor 4 has been found to contribute to this type of astrogliosis in a dry skin mouse model of itch [45]. Apart from astrocytes activation, spinal microglial activation has also been found in mouse models; for example, after intradermal injection of compound 48/80 (histamine-dependent) and 5′-guanidinonaltrindole [46]. Microglial activation can be subsided by intrathecal minocycline (a microglial modulator) which can reduce scratching and symptoms of dermatitis in a mouse model of AD [47]. 

In short, identification of peripheral and central components of itch [17,38] has pushed the field forward for novel and effective targeting. Several established models of itch exist which are applicable in both animals and humans. These models are useful in understanding itch pathways and also in testing novel antipruritics [48]. Many compounds are in early stages of development and several are going through final phases of antipruritic pipelines. 

BoNTs were initially used for muscle hyperactivity [49]. Soon after the identification of broader biological effects (e.g., neuronal and non-neuronal effects in dermal fibroblasts, sebocytes and vascular endothelial cells), additional indications garnered attention and further mechanisms underlying BoNTs effects were proposed [50,51]. The antipruritic effect of Botulinum Toxin Type A (BoNTA) was identified in an open-label pilot study of lichen simplex in 2002 [52]. Its antipruritic effect in dyshidrotic hand dermatitis was also reported in the same year [53,54]. Since then [6], BoNTs have been subjected to investigation for many other pruritic conditions, such as Hailey-Hailey disease and inversed psoriasis. First, one must understand how BoNTs can exert potential effects against itch. An acceptable rationale for application of BoNTs in itch is that acetylcholine mediates itch and BoNTA inhibits the release of acetylcholine from presynaptic vesicles. However, other mechanisms also play a role in antipruritic effects of BoNTs. In the section below, proposed underlying mechanisms of BoNTs in reducing itch are described. Since botulinum toxin type A (BoNTA) is the most used in the current literature, the rest of this manuscript focuses on this neurotoxin unless otherwise stated. 

## 3. Botulinum Toxin Type A (BoNTA)

BoNTA inhibits vesicular release of neurotransmitters by interfering with exocytotic release. BoNTA is composed of a heavy chain with a receptor-binding site and a translocation domain as well as a light chain with endopeptidase activity. This permits cleavage of synaptosomal-associated protein 25 (SNAP-25) which is an essential molecule for membrane fusion [55]. BoNTA was first known to block acetylcholine release at the neuromuscular junction [56]. It has been used for disorders with abnormal muscle contraction because of its ability to relax spastic muscles [49]. However, it became evident that BoNTA also inhibits the release of other transmitters, such as glutamate, substance P (SP), and calcitonin gene-related peptide (CGRP) [57]. The anti-itch effect of BoNTA is also a result of inhibition of acetylcholine release and other mediators involved in itch [8,9]. As such, evidence from analgesic properties of BoNTs in pain and nociception have also been inspirational for scientists [58,59,60,61,62,63,64]. 

## 4. Experimental Evidence for Antipruritic Effects of BoNTA in Healthy Humans

Our group was first to investigate the effect of subcutaneous administration of BoNTA on experimentally induced itch (histamine) in healthy subjects. Fourteen healthy men received BoNTA (5U, BOTOX^®^, Allergan, NJ, USA) on the volar forearm. Saline was used as control. Histamine prick tests were performed at the application sites before, one, three days, and a week after treatments. Itch intensity and neurogenic inflammation were evaluated. BoNTA significantly reduced histamine-evoked itch intensity, flare size, and vasomotor reactions to histamine [65]. 

Another study in healthy volunteers looked into the antipruritic effects of BoNTA in a non-histaminergic model where cowhage was used (clinicaltrials.gov; identifier: NCT02639052). In this study, 35 healthy subjects (16 men and 19 women; age 26.8 ± 6.8 years) were enrolled and intradermal BoNTA (10U, BOTOX^®^, Allergan, NJ, USA) was injected in a 4 × 4 cm area on volar arms. Saline was used as control. Itch intensity following application of cowhage was recorded before treatment and one week, one month, and three months post-treatment. BoNTA reduced cowhage-evoked itch at all time points, suggesting a long-lasting effect. This study was presented at the 9th World Congress on Itch [66]. 

## 5. Experimental Evidence for Antipruritic Effects of BoNTA in Animal Models

Animal studies have also been conducted to look deeper into the cellular-molecular mechanism(s) of antipruritic effects of BoNTA. 

It is generally accepted that TRPV1 (transient receptor potential cation channel subfamily V member 1) is essential for histamine-dependent itch [31], whereas TRPA1 (transient receptor potential ankyrin 1) is required for histamine-independent itch, e.g., chloroquine-evoked itch, bile acids-induced cholestatic itch [67], and oxidative stress-induced itch [68,69]. A mice study [70] has investigated the effects of BoNTA on acute and chronic itch and the possible association of TRP channels to antipruritic mechanisms of BoNTA. Findings from this study demonstrated that BoNTA inhibited chloroquine-evoked itch which is considered an acute non-histaminergic model similar to that of compound 48/80-induced itch. Compound 48/80 is a potent histamine-releasing agent, primarily from mast cells, with a subsequent depletion of tissue histamine [71]. It was also presented that, following a single intradermal injection of BoNTA (0.1 U) into the nape of the neck, mRNA expression of TRPV1 and TRPA1 notably decreased in DRG and lasted for seven days. Protein expression of TRPA1 was highly elevated following AEW (acetone–diethylether–water) treatment—a dry skin itch model—and pretreatment with BoNTA could significantly abolish upregulation of TRPA1 expression in this model. Authors proposed that TRPV1 and TRPA1 play an important role in both acute and chronic itch and that BoNTA might exert its anti-itch effects through downregulated expression of TPRV1 and TPRA1 in DRG [70]. This study confirmed that antipruritic effects of BoNTA present independently of mice models and can be used both for histamine-dependent and histamine-independent itch and dry skin-induced chronic itch [70]. 

Another study studied AD in mice models [72]. AD is accompanied by debilitating itch and a complex interaction is believed to exist between immune cells and nerve fibers [73]. NC/Nga mouse is a relevant animal model to study AD [74] because these animals spontaneously develop AD-like skin lesions under conventional conditions. In this study [72], the authors examined the protective effect of BoNTA (intradermal injection on the rostral back) on AD lesions in NC/Nga mouse. The primary outcome was skin thickness and transepidermal water loss. Authors assessed skin thickness, water loss, skin severity scores, histological alterations of skin, e.g., mast cell count, skin interleukin (IL)-4 mRNA and protein expression, and total serum IgE levels [72].This study showed that BoNTA could significantly suppress AD severity, IL-4 expression level, and the number of infiltrating mast cells [72]. Study period was limited to 14 days and long-term effects were not investigated. 

The effects of BoNTA on mast cell activity has also been studied in animal models [75,76]. In a study by Park [75], 10 Sprague Dawley rats were randomly divided into two groups receiving BoNT A and vehicle (control). A distally based 3 × 9 cm random pattern flap including the panniculus carnosus muscle was elevated. BoNTA was administered five days prior to flap elevation. Seven days after flap elevation, tissue samples (1 × 1 cm) were taken from the center of each flap. Findings [75] demonstrated that BoNTA decreased mast cell activity. 

Another animal study looked into the mechanisms of BoNTA in targeting psoriasis. In a KC-Tie2 mouse model of psoriasis [77], researchers showed that intradermal injection of BoNTA improved psoriasiform skin inflammation and epidermal hyperplasia. It also decreased the number of infiltrating CD4^+^ T cells and CD11c^+^ dendritic cells (DCs) in parallel with reducing the number of blood vessels and their adjacent nerves [77]. The decreased number of blood vessels within the affected skin of the treated mice illustrates the role of nerves and blood vessels in an inflammatory skin disease such as psoriasis. This study illustrates the role of blood vessel and nerve communication in psoriasis and the potential role of BoNTA in blocking this communication. Authors proposed that the persistence of some plaques in psoriasis patients might be explained by local microenvironments within the tissue, including nerve-derived SP and CGRP [77]. BoNTA, a known inhibitor of CGRP and SP release, can help with the interruption of this cascade and may present significant improvement in disease severity as early as two weeks after treatment. Therefore, they proposed that BoNTA may serve as a supplemental agent to topical or biologic therapeutic regimens [77].

## 6. Clinical Evidence for Antipruritic Effects of BoNTs

BoNTs have been used in clinics for many dermatological conditions that can present with or without itch. For a review, see A. Campanati et al., Y.S. Kim et al., and A.S. Al-Ghamdi et al. [7,8,78]. A recent review has summarized the use of intradermal BoNTA in treating chronic refractory pruritus based on 11 studies between 1996 and 2016 [79]. 

Many applications are still off-label [7] and the cases presented below both summarize current clinical evidence and encourage additional well-designed studies to reach a consensus on safe applicability, optimal dose, and delivery route for the standardization of BoNTs use for antipruritic effects.

### 6.1. Post Herpetic Itch

Post herpetic itch (PHI) is considered a type of neuropathic itch and has been investigated less than postherpetic neuralgia (PHN) [80]. PHN is a long-term neuropathic pain that remains after the rash from shingles (also known as herpes zoster) has healed. Varicella-zoster virus (VZV) is the cause of herpes zoster. Besides shingles, degenerative nerve root compression (notalgia paresthetica), and sensory polyneuropathy can cause neuropathic itch [81]. Almost half of PHN patients report PHI. This finding suggests that mechanisms underlying PHI and PHN are most likely independent [82]. PHI is a common disorder that equally affects men and women. PHI is age-independent and occurs in both young and old patients. PHI often appears on the head and neck (V1 dermatome) [82]. 

BoNTA treatment has been successful to reverse pain in PHN [83]. Accordingly, the usefulness of BoNTA in PHI has been considered [84] and the effectiveness of BoNTA for a neuropathic itch caused by dermatomal damage to the thoracic nerves has been presented [84]. In this study, BoNTA injections (dose range 16–25 U) were given in several points within the involved dermatome. Double-blind, randomized, control trials are required in a larger sample size before the use of BoNTA in different types of neuropathic itch such as PHI can be considered. BoNTs could be considered in severe cases of intractable PHI, which are not responsive to other options [80].

### 6.2. Brachioradial Pruritus

Brachioradial pruritus (BRP) was first described in Florida in 1968 by Waisman [85] and is classified as a deep itch of the forearms and upper trunk which can worsen with either scratching or sunlight [86]. Brachioradial pruritus is considered another neurogenic itch which often occurs in the upper extremities, usually localized on the dorsolateral forearm overlying the proximal head of the brachioradialis muscle; however, upper arms and shoulders may also be affected [87,88]. BRP might be unilateral or bilateral and it is still considered a common “tropical” dermopathy [89]. It is still not known if BRP is a symptom of neuropathy, similar to chronic cervical radiculopathy, or a condition that occurs secondary to chronic ultraviolet damage. Larger studies for better understanding of BRP are warranted. BRP responds to ice packs but efficacy is only temporary [90]. Lamotrigine and gabapentin have also been found useful for BRP. Intradermal injections of BoNTA (100 IU) was reported in a 59-year-old Caucasian female with BRP for 12 years [86]. This patient had disabling itch and a burning sensation on the upper posterior arms, scapular regions, and neck. A diverse range of topical and systemic treatments, hypnotherapy, and Chinese herbal medicine did not improve the patient’s condition. Application of icepacks was not beneficial in this case. However, this patient reported dramatic itch relief, lasting for up to six months, after four rounds of BoNTA injections. It was proposed that the effect of BoNTs for this condition and those similar to it, may have been due in part to its ability to block the release of neurotransmitters involved in itch, e.g., acetylcholine. 

### 6.3. Notalgia Paresthetica

Notalgia paresthetica (NP) [91,92] is a sensory neuropathic syndrome with pruritus, pain, paresthesia, hypo-hyper-esthesia, and burning as common symptoms. NP is characterized by a brownish itchy patch in the affected area. This condition mainly occurs in the elderly or in association with musculoskeletal disorders driven by spinal nerve compression, particularly at the C4–C6 level [92]. NP is a difficult condition to treat and quality of life is rather low in these patients. Less efficient treatments for NP are partially attributable to its unidentified underlying mechanisms or pathogenesis. 

In 2007, two NP patients were treated with BoNTA [84]. Later in 2010, Wallengren and Bartosik [93] reported limited effectiveness of BoNTA treatment in six NP patients. One double-blind randomized clinical trial for NP was reported in 2014 [94], in which the effectiveness of BoNTA was tested in 20 NP patients who were resistant to topical therapies. The study investigated pruritus, effects on hyperpigmentation, and global effectiveness as rated by both patients and investigators. Pruritus rated on VAS (visual analogue scale) did not show any itch reduction when it was compared between patients and controls (receiving saline) [94]. BoNTA treatment also did not improve hyperpigmentation or global efficacy indicators. In this study, injections of 0.1 mL (50 U/mL) for every 1–2 cm^2^ of hyperpigmented area were given. Maximum dose reached to 200 U [94]. 

Injection of BoNTA is an option, but further research is required to confirm safety and efficacy of BoNTA for NP. Patient selection and dose also need to be determined.

### 6.4. Lichen Simplex Chronicus (LSC)

Lichen simplex chronicus (LSC) is also known as neurodermatitis circumscripta. LSC is an eczematous dermatosis, characterized by intense localized pruritus and thickening of the skin with variable scaling arising secondarily from repetitive scratching or rubbing. This condition can be intense or recurrent and often disrupts sleep, sexual function, and quality of life in affected individuals. Breaking the itch-scratch cycle is challenging. Exact incidence in general population is unknown, but one study demonstrated that 12% of aging patients with pruritic skin presented with LSC [95]. This disorder is observed more commonly in females than in males. BoNTA has been considered an option for LSC [52]. One pilot study investigated the effect of intradermal injection of Abobotulinumtoxin A in five lesions in three patients and found that pruritus diminished within three to seven days in all patients. By four weeks, all lesions had cleared completely with no recurrences [96]. Another case study reported a successful result with BoNTA in a 55-year-old woman with a six-year history of intense facial pruritus at the right side of face [97]. Despite the small sizes of these studies, the antipruritic effects of BoNTA in LSC is promising [78]; however, additional large studies are required to confirm its efficacy in LSC.

### 6.5. Vulvodynia

Vulvodynia is a complex disorder [98] affecting 16% of women in the general population. It is described by burning, stinging, itching, irritation, or rawness. The International Society for the Study of Vulvovaginal Disease (ISSVD) has defined vulvodynia as “vulvar pain occurring in the absence of an underlying recognizable disease.” There are no clinical or histopathologic criteria for the diagnosis other than consideration and careful evaluation to exclude other causes of pain. Successful therapy often requires a multidisciplinary approach with more than one type of therapeutic intervention. BoNTA has been shown to be effective treatment for vulvodynia [99]. While several small open-label studies have shown improvement in symptoms with botulinum toxin at doses of 20–100 units, the only randomized double-blind, placebo-controlled trial demonstrated no improvement with 20 units over placebo in 64 women with vulvodynia [100].

### 6.6. Keloids and Hypertrophic Scars

Keloids and hypertrophic scars are structures formed during the wound healing process and present with dysregulated growth and a high level of collagen formation. To prevent these scars, silicone dressings, laser therapy, and immune response modulators are applied [101]. Intralesional (IL) corticosteroid therapy with triamcinolone acetonide is a common therapy in keloids treatment [102]. In 2000, Gassner and colleagues [103] suggested that BoNTA injections can paralyze muscles close to wounds and subsequently reduce pressure on wound edges. This first study was conducted in a primate model and confirmed the hypothesis. Another study used optical 3D profilometry as an objective evaluation of keloids following treatment by BoNTA [104]. Only four patients were included in this study and no changes was evident on fibroblast proliferation. In a rabbit ear hypertrophic scar model [105], BoNTA also was found to have less effect on hypertrophic index, fibroblast density, and collagen density when it was compared with IL triamcinolone acetonide and 5-fluorouracil [106]. However, other in vitro and experimental animal models support BoNTA as treatment of keloids and scars [107]. BoNTA delays fibroblast growth through the inhibition of the cell cycle which subsequently reduces hypertrophic scar development. BoNTA also decreases the expression of connective tissue growth factor and inhibits the growth of fibroblasts and scar expansion. BoNTA reduces the concentration of TGF-β1 in fibroblasts and decreases the infiltration of inflammatory cells during wound healing; it also reduces fibrosis [107]. 

In 2015, a large randomized double-blind study tested the effect of BoNTA compared with IL corticosteroid therapy in 24 patients with keloids [108]. In this study, patients were allocated to receive IL steroid every four weeks for six sessions and IL BoNTA 5 IU/cm every eight weeks for three sessions. Hardness, elevation, and redness, together with itching, pain, and tenderness were evaluated and patients were asked for their subjective satisfaction. No significant difference was observed between groups in most of the measured parameters. However, patients receiving BoNTA reported higher satisfaction with their therapy. Authors proposed that BoNTA might have reduced small-fiber neuropathy causing itching, pain, and allodynia [108]. 

A potential use for BoNTA in keloids and hypertrophic scars is predicted. But additional randomized double-blind controlled trials are needed to compare with current treatments to evaluate efficacy and safety profile. Efficacy in the prevention and treatment of hypertrophic scars might vary according to the scar’s location on the body; hence, testing both facial and other body parts such as chest or back is proposed. Surgical and trauma wounds must also be differentiated. In addition, stratification according to ethnicity and age is essential as both elements affect wound healing. 

### 6.7. Psoriasis

Psoriasis is a skin disorder strongly linked to both genetic and environmental factors. An immunological reaction mediated by T lymphocytes is thought to be the main player in the pathogenesis of psoriasis. Cutaneous inflammation and keratinocyte hyperproliferation are featured characteristics of such a response. 

Inverse or flexural psoriasis is a specific form of psoriasis with red, dry, smooth, and shiny skin. Clinically, inverse psoriasis manifests with sharply demarcated erythematous plaques with infiltration that accompany sensations of itching and burning. Common locations of inverse psoriasis include armpits, groin, under the breasts, and in other flexion skin folds, such as around the genitals and buttocks. It is particularly troublesome for patients with deep skin folds and/or those who are overweight. Treatment of inverse psoriasis can be difficult. Steroid creams and ointments are considered effective; however, overuse of steroids can result in side effects, especially thinning of the skin and stretch marks. Skin folds, where inverse psoriasis is common, are susceptible to yeast and fungal infections. Topical immunomodulators, such as tacrolimus and pimecrolimus, have also been effective. 

Administration of BoNTA has been proposed as a novel therapy in inverse psoriasis [109,110] in consideration of its mechanism of action in the neuroglandular junction, which reduces sweating. However, a link between high nerve fiber density in psoriatic skin and elevated CGRP and SP release has been reported. It has been demonstrated that psoriasis undergoes remission phases as a result of innervation loss or lack of nerve function, such as following a nerve injury. This can explain how BoNTA inhibits CGRP and SP release from nerve endings and can lead to subjective reports of improvement after administration of BoNTA. 

Zanchi and colleagues [109] reported that in 15 patients with inverse psoriasis, BoNTA presented effectiveness; however, the effect was mainly evaluated by self-assessment in patients rating itch and pain on a visual analogue scale (VAS). In this study, psoriasis that was located in the armpits, submammary sulcus, intergluteal folds, inguinal folds, and umbilicus in patients was treated with BoNTA injections with a total dose of 50–100 U per patient relative to psoriasis extent and severity. Evaluations were performed before and after treatment in weeks 2, 4, and 12. The erythematous area was defined using objective photographic evidence and subjective patient assessment of pain and itch was assessed using a 10-point VAS. BoNTA therapy resulted in improvements in subjective patient symptomatology and objective reductions in erythema and maceration in the treated areas according to photographic evidence. However, findings from this study were questioned [111], pointing to the lack of quantitative assessment for improvement; for example, using psoriasis area and severity index (PASI) or obtaining histological evident before and after the treatment. 

Overall, current evidence demonstrates that BoNTA is capable of reducing pain, itch, and inflammation in psoriasis-affected skin. Dermal and epidermal cytokines and peptides produced by keratinocytes, fibroblasts, lymphocytes, and macrophages are involved in the pathogenesis of psoriasis. Interleukin-1 (IL-1) stimulates the proliferation of keratinocytes and the production of cellular adhesion molecules which then stimulate the release of other cytokines (e.g., IL-6, IL-8). IL-6 stimulates the proliferation of B and T lymphocytes, which is an important factor in stimulating keratinocyte growth, and IL-8 exerts a powerful chemotaxis action towards leucocytes. In future randomized clinical trials evaluating the potential role of BoNTA in the treatment of psoriasis, special attention needs to be given to psoriasis as a variable pathology with several spontaneous relapses and remissions over time which can cause difficulties in evaluation of effectiveness. Amount and depth of injection are yet to be determined. Assessment of effectiveness must include both subjective and objective parameters including cutaneous sensory, vasomotor, and autonomic function. Safety, tolerability, and cost effectiveness should also be carefully evaluated before considering BoNTA as a routine clinical practice. 

### 6.8. Pompholyx

Dyshidrotic eczema, also called pompholyx, is a common relapsing vesicular-bullous disease found on the palm or soles of feet [112]. The pathogenesis of this condition is still unresolved; however, one study examined the roles of aquaporin 3 and aquaporin 10, which are water channel proteins located in the epidermis, and concluded that overexpression of these channels may play a role [113]. Wet works, sweating, and occlusion are among the provoking factors. Pain, itch, and burning sensations together with discomfort in wearing gloves or shoes, bacterial infection, or mycosis are among the common symptoms. 

An improvement in hand eczema was observed in patients with palmar hyperhidrosis following intradermal BoNTA [53]. This study was conducted in 10 patients with bilateral vesicular hand dermatitis where BoNTA injections (100 U BOTOX^®^) or saline (control) were given in either hand. Seven out of the 10 patients reported a good or very good effect of the treatment. Another study [54] applied topical corticosteroids on both hands in combination with intracutaneous injections of BoNTA (100 U BOTOX^®^) in six patients with more severely affected hands. A rapid improvement in pruritus and vesiculation was observed in the treated hand with combination therapy. A case study [114] has also demonstrated BoNTA effects in palmar pompholyx. No placebo-controlled trial is available. Therefore, effective and safe application of BoNTA for dyshidrotic eczema requires further validation.

### 6.9. Postburn Itch

Itching is a common secondary symptom related to burn injuries [115]. Research has proposed multiple mechanisms underlying itch as secondary to burn conditions. Several medications have been identified and used to manage this condition. BoNTA has also been considered as an option. In 2012, a study was conducted [116] to investigate the effectiveness of BoNTA and found that 87.5% of patients rated their postburn itch as severe (>7). Following the administration of BoNTA, itch intensity dropped to zero within four weeks. The average duration of the symptom-free period was nine months (range 3–18 months). BoNTA might be an option for burn-associated itch which are resistant to conventional therapies. This study [116] only included a small sample size and larger studies are warranted before establishment of this treatment at clinic. 

### 6.10. Fox–Fordyce Disease

Fox–Fordyce disease (FFD), characterized by intensely pruritic papules in apocrine gland-bearing regions, is a rare disorder for which there is currently no definitive treatment or known cure [117]. FFD is a chronic, pruritic disorder caused by keratin plugging of the follicular infundibulum at the distal portion of the apocrine sweat duct and less often by plugging of apoeccrine ducts. This obstruction causes apocrine sweat retention and, over time, rupture of glands with secondary inflammatory dermal alterations. The etiology remains unclear although epidemiological data support a hormonal component, as women between 15 and 35 years of age are more commonly affected and this condition may remit after menopause. 

The condition is often intensely pruritic and is usually associated with hypohidrosis. Pruritus is aggravated by emotional, physical, or pharmacological stimulations that enhance sweating. Therapeutic knowledge of FFD is derived from case reports but no large case series has been carried out. Topical and intralesional corticosteroids are often a first-line therapy. In medication-refractory cases, surgical interventions have proven to be successful. A case has been presented in which BoNTA injections resulted in the disappearance of pruritus and a partial clinical response after one session [117]. This response was sustained over time. The study’s authors suggested that chemodenervation of cholinergic nerve terminals to the eccrine and apoeccrine glands, inhibiting their sweat secretion, might be considered as underlying mechanism for the effects seen in this case. Other cases of hyperhidrotic pruritic axillary granular parakeratosis responders to BoNTA have also been reported [118]. Clinical trials to evaluate optimal treatment regimen with BoNTA for FFD are required.

### 6.11. Hailey-Hailey Disease

Hailey-Hailey disease (familial benign pemphigus) is a rare genetic skin disease, often presented with blisters or vesicles and erythematous plaques in skin folds. Axilla, groin, neck, and inframammary folds are amongst the most common sites of disease manifestation. The disease’s associated red scaly areas can be itchy. The topical and oral corticosteroids, oral retinoid, cyclosporine, and methotrexate used for treatment are often linked to side effects. There are cases of Hailey-Hailey disease treated with BoNTA with successful outcome [119,120]. Reduced sweating and local irritation help to improve lesions conditions as well as a reduction in itch. BoNTA can potentially be considered as a treatment option, in particular for those patients with limited response or intolerance to other treatments [120].

### 6.12. Rhinitis

Rhinitis is an inflammation of the nasal mucous membranes, presented with nasal discharge, nasal obstruction, sneezing, and itching [121,122]. It is a common disease affecting around 20% of general population and can be divided into infectious, allergic, occupational, drug-induced, hormonal, and idiopathic rhinitis (IR). The latter is also known as non-allergic, noninfectious perennial rhinitis, intrinsic, or vasomotor rhinitis. BoNTs have been used in both allergic [123] and idiopathic rhinitis (IR) [124,125]. 

Allergic rhinitis (AR) is a noninfectious inflammatory disorder in nasal mucosa provoked by an allergen exposure and an IgE-mediated immune response. The major mediator of nasal inflammation in AR is histamine which causes symptoms such as vascular permeability, mucus secretion, and stimulation of the sensory nerve fibers. Other mediators involved are neurokinin A, SP, CGRP, VIP, and neurotrophins. Current treatments include intranasal corticosteroids, antihistamines, mast cell stabilizers, and leukotriene receptor antagonists. AR can benefit from BoNTA injection, when it is given intranasally [124]. No serious adverse or systemic effects have been noted but burning after injection, nasal dryness, and epistaxis have been recorded. Nasal injection of BoNTA has shown comparable therapeutic effect to cetirizine in AR [126]. Arguments for the use of BoNTA in rhinitis are grounded in its modulatory effect on the secretory tone, which is related to the action of autonomic nervous system. A similar rationale has been used for the positive effects of BoNTA in Frey syndrome, hyperhidrosis, and sialorrhea. AR’s symptoms result from the activation of inflammatory mediators and an imbalance in the autonomic nervous system. Histamine, prostaglandin, and leukotrienes enhance vascular permeability and produce edema, in addition to altering the balance of the autonomic nervous system [127]. Underlying mechanisms of IR remain to be elucidated; however, autonomic nervous system imbalance with a dominant parasympathetic tone in the nasal mucosa has been proposed. Nasal blockage and rhinorrhea are more common in IR, while itching and sneezing are mostly present in AR. All symptoms can be prevented by application of BoNTA. 

Several other mechanisms for BoNTA effect in nasal mucosa have been proposed [124]. For example, BoNTA can induce apoptosis in the nasal glands, inhibit acetylcholine release from nasal mucosa nerve endings, decrease the release of neuropeptides (e.g., VIP and SP) from the trigeminal and parasympathetic nerve endings, and inhibit acetylcholine release from preganglionic cholinergic nerve endings in the sphenopalatine ganglion. As such, it has been suggested that targeting an upstream source of parasympathetic innervation at the sphenopalatine ganglion can potentially affect both nasal mucosa and nasal glands to yield an additive effect from the intraganglionic injection. A study proposed a technique to inject BoNTA into the posterior lateral nasal wall, which is located adjacent to the sphenopalatine ganglion. They conducted a pilot study with this technique applying a low dose of 25 units, resulting in only moderate discomfort to participants but yielding safe and effective results. Improved effects on congestion and itch had already been seen with dosages above 12 units. Accordingly, the authors suggested that low dose administration of BoNTA can be advantageous from a safety perspective.

BoNTA also may be considered in patients resistant to other treatments or intolerant to current treatments, e.g., nasal corticosteroids or systemic antihistamines. This treatment also allows for longer lasting effects, beneficial for patients. However, larger studies are required to identify the long-term effects and safety profile of BoNTA [126]. Further investigation is needed to identify whether BoNTA is to be used in clinic for rhinitis and which technique would yield the optimal outcome (e.g., posterior injection, turbinate injection, septal injection, or topical) [128]. RT001 is a novel topical gel formulation which contains a purified 150kDa BoNTA protein that has been used in a rat model [129]. The gel formula includes a prietary peptide to enhance transcutaneous and transmucosal flux of BoNTA. In the model, after a single intranasal administration of RT001, associated clinical signs of rhinitis, including inflammation, were significantly resolved within 5 days after treatment. 

The optimal dose and patient selection also need to be determined. In addition, it is still unclear whether and how repetitive administration of BoNTA would influence the outcome. Desensitization following repeated application is still an open question. Another important point is that some outcome measures are difficult to be objectified, for instance, nasal pruritus. Hence, analyzing effectiveness calls for the development of some objective methods to complement existing subjective instruments.

## 7. Concluding Remarks and Future Perspectives

This review highlighted the potential for BoNTs with a major focus on BoNTA to relieve pruritus. A lack of a sufficient number of randomized controlled trials, limited sample size in the current literature, diverse range of outcome measures, and a lack of knowledge about placebo effects make it difficult to draw a firm conclusion on the antipruritic effects of BoNTs. In addition, for each condition, several critical components remain unidentified, including safe and effective dosage, route of delivery (also considering new formulations of BoNTs), single versus repeated application with optimal interval, and standardization of techniques used in outcome measures. In addition, a strategy for patient selection and precise identification of responders in terms of gender, age, and ethnic background would substantially aid in targeting the right group for optimal effect. The long-lasting effects of BoNTs make it desirable in terms of patients’ compliance. However, measured use must also be considered in terms of cost and comparable effectiveness with other agents available for each pruritus condition. Most of the studies presented in the literature have suggested BoNTA as an option; however, not as a first-line therapy and predominantly for those patients either who are having recurrent problems or who are non-responders to other treatment options. It is likely only a matter of time before the full potential of BoNTs for pruritus is elucidated. However, for the time being, focus should be on more common conditions or those for which stronger evidence exists for successful use of BoNTs can be on those conditions that are more common and stronger evidence exist in the literature for successful use of BoNTs. 
